# Addressing Single Nuclear Spins Quantum Memories by a Central Electron Spin

**DOI:** 10.1007/s00723-022-01462-2

**Published:** 2022-02-11

**Authors:** V. Vorobyov, J. Javadzade, M. Joliffe, F. Kaiser, J. Wrachtrup

**Affiliations:** 3rd Institute of Physics, Center for Applied Quantum Technologies and Institute for Quantum Science and Technology, University of Stuttgart, Stuttgart, Germany

## Abstract

Nuclei surrounding single electron spins are valuable resources for quantum technology. For application in this area, one is often interested in weakly coupled nuclei with coupling strength on the order of a few 10–100 kHz. In this paper, we compare methods to address single nuclear spins with this type of hyperfine coupling to a single electron spin. To achieve the required spectral resolution, we specifically focus on two methods, namely dynamical decoupling and correlation spectroscopy. We demonstrate spectroscopy of two single nuclear spins and present a method to derive components of their hyperfine coupling tensor from those measurements.

## Introduction

In the past, spins have been mainly used in spectroscopy, as they are a highly sensitive and indicative probe for their environment. Both in EPR and NMR, spin resonance experiments are constantly generating a valuable amount of information in material science, chemical and biochemical analytics as well as medical diagnostics. The success of both methods is based not only on the rich structural and dynamical information they can acquire, but also on the outstandingly good coherent control of electron and nuclear spins, which enable sophisticated pulse sequences. A wealth of methods both in EPR and NMR have been developed to acquire high spectral resolution or use coherence as well as polarization transfer to unravel complex spectra [1].

In the past few years, these excellent quantum control properties have brought electron as well as nuclear spins into the focus of quantum technology research. In this field, highly controllable quantum systems are used to generate quantum states on demand, being of use in quantum sensing, -communication and -computing [2, 3]. The specific requirements for a system to qualify as a good quantum bit (qubit), besides very good coherent control, are long relaxation and coherence times, addressability as single spin, single quantum state readout and controlled qubit–qubit interaction. While spins typically fulfill the first two requirements, they are notoriously difficult to address as single spins and it is very hard to perform non-destructive state readout and generate controlled interactions. This is especially true for solids, where electron spins interaction with phonons or with uncontrolled impurity spins dominate relaxation behavior, very much in contrast to ions in vacuum [4]. As a result, only very few systems have matured into fully fledged optically addressable spin quantum bits. Among those, dopants in wide band gap semiconductors are the most investigated ones. Dopants in materials like diamond, silicon carbide, gallium nitride or boron nitride can be selectively addressed by optical means, show unpaired electron spins with, in part, spectacular coherence and relaxation times as well as excellent coherent control [5, 6]. Attempts to achieve controlled entanglement among multiple electron spins are underway and different schemes have been explored. Electron spins are brought into close contact such that their dipole–dipole interaction is large enough to generate entangled states with high fidelity [7]. As mutual electron–electron distances need to be smaller than 50 nm, such that the interaction strength is large enough to allow for efficient entanglement generation within the coherence time, this requires sophisticated nanopositioning techniques [8]. A method specific to systems which show coherent electron spins as well as emission of coherent photons is to achieve entangled electron spins by photon emission [5]. In this approach, single, identical photons emitted from two single dopants are brought to interference on a beam splitter, which under certain circumstances renders the spins of the dopants to be entangled. This scheme has been successfully employed to demonstrate entanglement of electron spins over distances larger than 1 km [9]. Irrespective of the type of coupling evoked among the electron spin, to achieve a deterministic high-fidelity entanglement, long lived quantum memories are needed to make large scale entanglement possible [10]. This quantum memory, in most cases, are nuclear spins in the surrounding of the electron spin [11]. Because of the high-fidelity control of nuclear spins, they can be used not only as memory but one can also use them as model system for small scale quantum registers. Specifically, nuclear spins have been used in a number of intriguing demonstrations, ranging from few qubit algorithms [12] to a ten qubit register with entanglement [11] and minute long coherence time [13] as well as first steps towards fault tolerant gates [14].

**Fig. 1:**
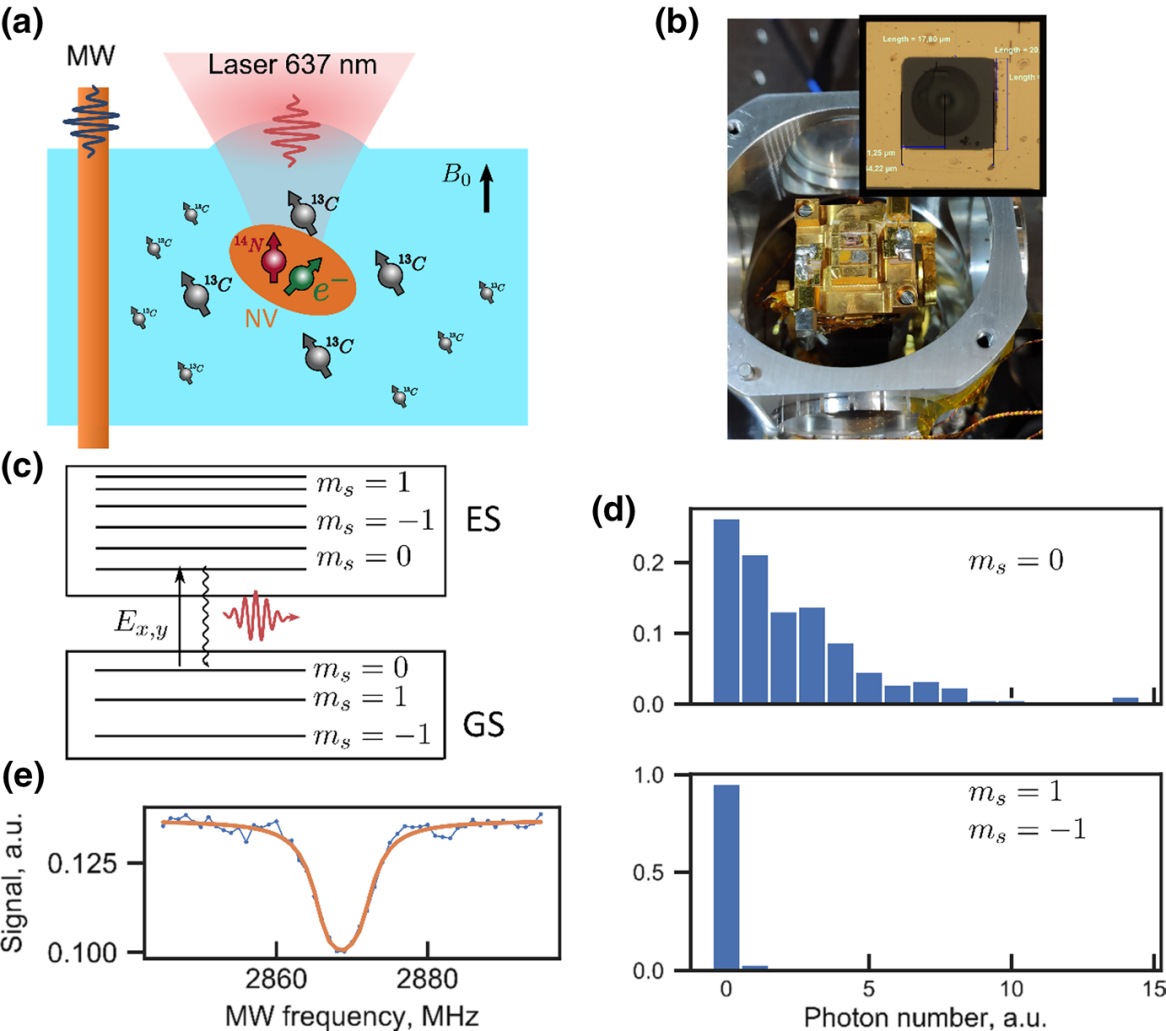
**a** Schematics of the experiment, showing a single NV center electron spin and its nuclear spin environment comprising ^13^C as well as ^14^N. **b** Picture of the sample chamber as well as of the diamond sample with the solid immersion lens used to enhance the light extraction efficiency of the NV center. **c** Simplified energy level diagram of the NV center showing its triplet ground—as well as first excited state. The data in **d** are taken under resonant excitation of the *E*_*x*,*y*_ transition. **d** Photon counting histogram with the spin being in the ${m}_{\mathrm{s}}=0$ or ${m}_{\mathrm{s}}=\pm 1$ state. **e** The optically detected magnetic resonance of NV center at zero magnetic field with off-resonant 520 nm laser excitation

The quality of these nuclear spins in terms of their use in quantum technology strongly depends on the environment of the electron spin they are coupled to. If this environment contains only a small number of non-zero nuclear spin species, single nuclear spins can be addressed by their distance and orientation dependent hyperfine coupling [15, 16]. Various electron–nuclear spin systems have been investigated in this regard. Among those are molecular systems with paramagnetic ground [17] or metastable states [18] as well as a wealth of solid-state systems in various host materials, such as diamond or silicon carbide. A specific, well-studied example is the nitrogen-vacancy center in diamond [19]. The center comprises a substitutional nitrogen with a next nearest neighbor vacancy embedded in the diamond carbon lattice. It shows a strong dipole-allowed optical transition between the electronic ground and first excited state at a wavelength of 637 nm with a phononic sideband, resulting in an efficient radiative transition. The center is a spin triplet (*S* = 1) state in both the ground and excited state. As the natural concentration of *S* = 1/2 nuclear spins in diamond is around 1%, few enough nuclear spins couple to a single electron spin to show single nuclear spin specific hyperfine couplings. These nuclear spins serve as memory quantum bits. Figure 1 shows the experiment schematically. A single NV center is selected in a focused laser beam (see Fig. 1a). All experiments are carried out at temperatures *T* ∼ 6 K such that the sample, its positioner as well as microwave coupling structures are mounted in a cryostat (Fig. 1b). As we detect spin signals optically, no resonant microwave structures are used. In addition, as we are working with single spins, static as well as MW magnetic field inhomogeneities are irrelevant. As a result, a single wire suffices to couple microwave irradiation to the sample. To achieve sufficiently large field amplitudes, the wire is placed close to the electron spin under study (a few 10 μm), which yields electron spin rabi frequencies of up to 100 MHz [20]. The signal-to-noise ratio in our experiments is determined by the number of photons received by the detector. Due to the large refractive index of diamond; however, most of the photons emitted by the NV defect are reflected from the surface. To be able to extract as many photons from the NV center as possible, a solid immersion lens (SIL) is fabricated around the defect (see inset of Fig. 1b) [21].

Optical detection of electron- and nuclear spin resonance commences as follows. At temperatures below 10 K, the optical transitions between the ground and first excited state get spectrally narrower than the energetic spacing between spin sublevels, which are split due to their zero-field splitting (around 2.8 GHz, see below) as well as Zeeman interaction with the external magnetic field. Owing to the small spin–orbit coupling, this excitation is spin-conserving, i.e., no change in the spin ${m}_{\mathrm{s}}$ quantum number occurs in first order. A laser resonant with the energetically lowest optical transition excites the center from one of its triplet ground state sublevels to a corresponding level in the excited state of the system. If the MW are resonant with the spin transition in the ground state, the NV center does not absorb the laser anymore and hence does not emit photons. Figure 1e shows the corresponding ODMR spectrum as a reduction in fluorescence intensity upon spin resonance. Only a single line is seen as the system is a ground-state spin triplet with *E* = 0 (see discussion below).

It is worth mentioning that the system allows to go one step beyond mere detection of electron spin resonance. If the photon rate is large enough, the quantum state of the spin can be determined faster than electron spin relaxation occurs, i.e., a *T*_1_ induced spin quantum jump. As a result, the electron spin quantum state can be determined by the emission intensity. Figure 1d shows a corresponding example. The figure depicts a photon number histogram in which the probability that a specific number of photons is detected within a given time interval. As the experiment clearly shows, the detected number of photons is markedly different for the spin to be in ${m}_{\mathrm{s}}=0$ or $1$. Setting a threshold on the photon counts allows us to discriminate between states [22]. In the case of the example shown in Fig. 1d, this threshold would be, e.g., at 5 photon counts. This quantum state readout capability is an important requirement for quantum information processing, for example in error correction algorithms.

**Fig. 2:**
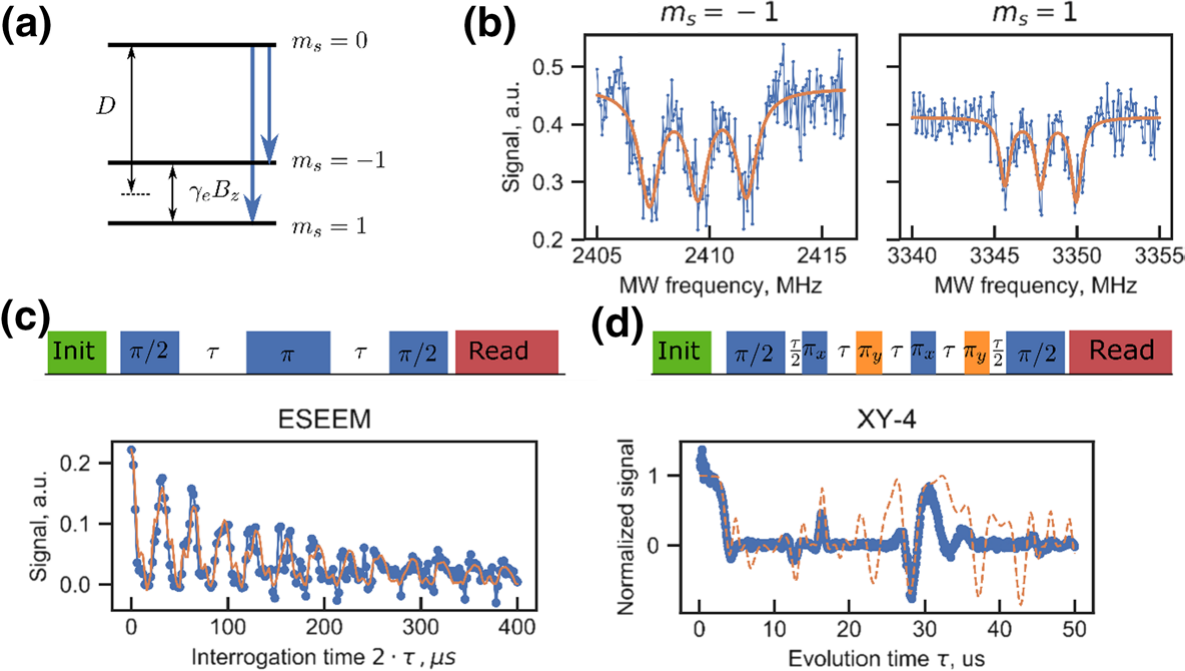
Optically detected magnetic resonance of a single NV center. **a** Spin level diagram showing the spin triplet ground state in a small (56 G) magnetic field aligned along the symmetry axis of the defect. **b** Optically detected magnetic resonance spectrum of the NV center showing ^14^N hyperfine coupling. **c** Two pulse Hahn echo on the defect with *π* pulse lengths of 29 ns. The data panel shows the echo amplitude as a function of waiting time with indication of a strong spin echo envelope modulation (ESSEM). The fit model presents an ESEEM analytical solution for two ^13^Cs with fixed hyperfine coupling found from correlation spectroscopy and four other nuclear spins as fitting parameters **d** same NV center subject to an XY-4 decoupling sequence. Dashed line is the simulation of XY-4 sequence with nuclear spins found from the fitting procedure of Fig. 2c

Next, we focus on addressing single nuclear spins in the environment of the electron spin. The experiments shown in Fig. 2 have been carried out in an external magnetic field of ~ 58 G. At this field, the electron spin resonance energies are determined by its zero-field splitting:

$${H}_{\mathrm{s}}=SDS+ {\gamma }_{\mathrm{e}}{B}_{0}S$$

with the zero-field splitting $D$ being on the order of 2.87 GHz. For low stain samples [23], as is the case for the defects used in this study, the *E* parameter is zero, i.e., the defect shows perfect C_3v_ symmetry. The magnetic field is aligned along the symmetry axis of the NV defect (*z* axis). In the case of small ${B}_{0},$ the splitting between the two states ${m}_{\mathrm{s}}=\pm 1$ is given by ${\gamma }_{\mathrm{e} }{B}_{z}$. Figure 2b shows the electron spin resonance spectrum of the ${m}_{\mathrm{s}}=0$ to ${m}_{\mathrm{s}}=\pm 1$ transition, recorded by observing the NV center fluorescence intensity. The line structure is dominated by the ^14^N hyperfine and quadrupole coupling, resulting in a 2.16 MHz almost symmetrically split triplet. In CW spectra, the line splitting only becomes observable with a carefully aligned magnetic field and low enough strain. In this case, the hyperfine coupling is a first order perturbation to the electronic spin Hamiltonian and the ^14^N hyperfine coupling is clearly visible. The ^14^N nucleus has been investigated as a quantum bit and its potential use as quantum memory. Its large coupling allows for fast operations, e.g., quantum gates like a swap of an electron spin quantum state to the ^14^N nuclear spin. However, the strong coupling also leads to an unwanted side effect. If the defect center is excited optically, it is promoted from its electronic ground state to the first excited states. Both states have entirely different symmetries (2A in the ground state and E in the first excited states). This entails rather large changes in the spin density distribution. In the ground state, the electron spin density at the nitrogen nucleus is 2%, while it is around 60% in the first excited state [19]. As a result, the hyperfine coupling increases from 2 to 60 MHz in the excited states. The excited state relaxes spontaneously to the ground state, and hence together with the large change in the hyperfine coupling causes a very fast dephasing of the ^14^N spin quantum state. The nucleus is thus not a good quantum memory for defects which are optically probed, like it is the case in quantum networks.

Because of the low abundancy of paramagnetic nuclei in diamond (1.1% ^13^C) and the fact that we are probing a single electron spin, one would expect to see hyperfine coupling to ^13^C nuclei also in CW spectra. Indeed, this is the case when a ^13^C nuclear spin is found in the first or second coordination shell around the defect center, such that the hyperfine coupling exceeds a few megahertz [24]. However, those nuclei entail identical challenges compared to the ^14^N spin. Because of the large changes in the ground to excited state spin density distribution, they dephase rather fast upon optical excitation. The only way to mitigate this process is to resort to less coupled nuclei.

There are several known ways to extract small hyperfine couplings beyond the limit set by *T*_2_^*^. One particularly easy way of doing so, is to perform a two-pulse echo on the electron spin and observe nuclear spin induced quantum beats. As the echo decays on the time scale of *T*_2_ (around 200 μs for the present case), much smaller couplings can be resolved. An example of such an experiment is shown in Fig. 2c. The experimental procedure follows a standard Hahn echo (HE) pulse sequence with a *π*/2 pulse length of 14.5 ns and a *π* pulse of a 29 ns. For optical detection, a final *π*/2 pulse is necessary to convert spin coherence into ${m}_{\mathrm{s}}=0$ population, which is then read out by the method described in Fig. 1. Figure 2c shows a representative trace of such an experiment, where the echo intensity is plotted as a function of waiting time *τ*. A strong echo modulation is visible with a periodicity of around 33 μs. This coincides with the Larmor precession of ^13^C nuclei at the applied magnetic field $(\approx 60 \mathrm{kHz})$. The occurrence of a modulation at this frequency is specific for a *S* = 1 systems, as in the *m*_s_ = 0 sublevel no hyperfine coupling occurs and hence all nuclei in the sample process with identical frequency. On top of the revival pattern, we see ESEEM (electron spin echo envelope modulation) features originated from individually coupled nuclear spins as additional modulation. However, because of the strong echo modulation from the free precession, this modulation pattern proves to be not sufficient to derive hyperfine coupling parameters. A strategy to resolve these hyperfine couplings is to employ higher order decoupling sequences which lengthen the electron spin coherence time even further and hence allow for the resolution of even smaller hyperfine couplings. These schemes have been worked out earlier in the context of single spin detection and should be used here to discuss the detection of hyperfine couplings down to a few kilohertz.

While ESEEM-based hyperfine spectroscopy is standard in ESR, this is not so much the case for higher order decoupling sequences, like shown in Fig. 2d. Dynamical decoupling sequences allow for the detection of weakly coupled individual nuclear spins embedded within the spin bath, as they provide high sensitivity for weak signals and effectively filter out background noise. Progressively higher order sequences reduce the interaction of the spin bath and hence increase coherence times. Timing the pulse spacing to be on resonance with the dynamics of the system allows a larger electron phase to be accumulated and hence increases the signal. Sequences like XY4 used in Fig. 2d vary the *π* pulses between the *X* and *Y* axes to correct for off resonance and pulse length errors that can accumulate in many pulse sequences. The function of these pulse sequences to detect nuclear spins is best seen by considering that the signal detected by the electron spin is proportional to the phase *Φ* it acquires during the pulse sequences. This accumulated phase is given by [25]

$$\Phi ={\int }_{0}^{t}\gamma {B}_{\mathrm{nuc}}\left({t}^{^{\prime}}\right)y\left({t}^{^{\prime}}\right)\mathrm{d}{t}^{^{\prime}},$$

where $y\left({t}^{^{\prime}}\right)$= ± 1 is the modulation function of the sequence that changes sign whenever a *π* pulse is applied. *B*_nuc_(*t*) is a harmonic function, because the Larmor precession of the nuclear spins generates a periodically modulated magnetic field at the location of the electron spin. In this case, the phase is given by [25].

$$\phi =\frac{\gamma {B}_{\mathrm{nuc}}^{a}}{2\pi {\nu }_{\mathrm{ac}}}\left[\mathrm{sin}\left(\alpha \right)-{\left(-1\right)}^{n}\mathrm{sin}(2\pi {\nu }_{\mathrm{ac}}t+\alpha \right)+2\sum_{j=1}^{n}{(-1)}^{j}\mathrm{sin}(2\pi {\nu }_{\mathrm{ac}}t+\alpha ).$$

Here *α* is the phase of the signal to be detected, *ν*_ac_ is its frequency, and t is the time over which the signal from n coupled nuclei is accumulated. This can be formulated in a more compact form as.

$$\phi =\gamma {B}_{\mathrm{nuc}}^{a}tW({\nu }_{\mathrm{ac}},\alpha ).$$

Here, $W({\nu }_{\mathrm{ac}},\alpha )$ defines a weighting function which is specific for the pulse sequence used. This weighting function has peaks at *ωτ* = (2 *k *− 1) *N* *π*, *k* = 1, 2, … For weakly coupled spins and high B fields, where the Larmor frequency is much larger than the coupling frequency, these peaks cause an apparent loss of coherence at the corresponding frequencies in the dynamical decoupling measurements. The more decoupling pulses can be used, the weaker coupled spins can be detected. The weighting function predicts that the intensity of the coherence dips increases as *N* [2, 26].

If the nuclear spins are not weakly coupled, i.e., their inverse coupling strength is larger than the decoherence time, the electron and nuclear spins interacts coherently. As a result, the electron and nuclear spins become entangled throughout the pulse sequence. An indication for this is seen in Fig. 2d, where the signal becomes zero, such that the traced-out electron spin state is an effective thermal state. In this case, assignment of certain peaks in response to the XY4 sequence to specific values of the hyperfine coupling becomes less obvious. In addition, if the spectral density of couplings is large, individual peaks cannot be resolved any longer.

The technique has been very successfully used to isolate the coupling to weakly coupled nuclear spins, i.e., those spins in which the hyperfine coupling is smaller than their Larmor frequency [26]. However, the technique is not well suited to do spectroscopy on stronger coupled spins, such as the one used here. Mostly, this is because strong coupling requires short pulse spacings and second, because the spin evolution does not correspond to classical noise but rather comprises strong components due to coherent spin evolution.

**Fig. 3:**
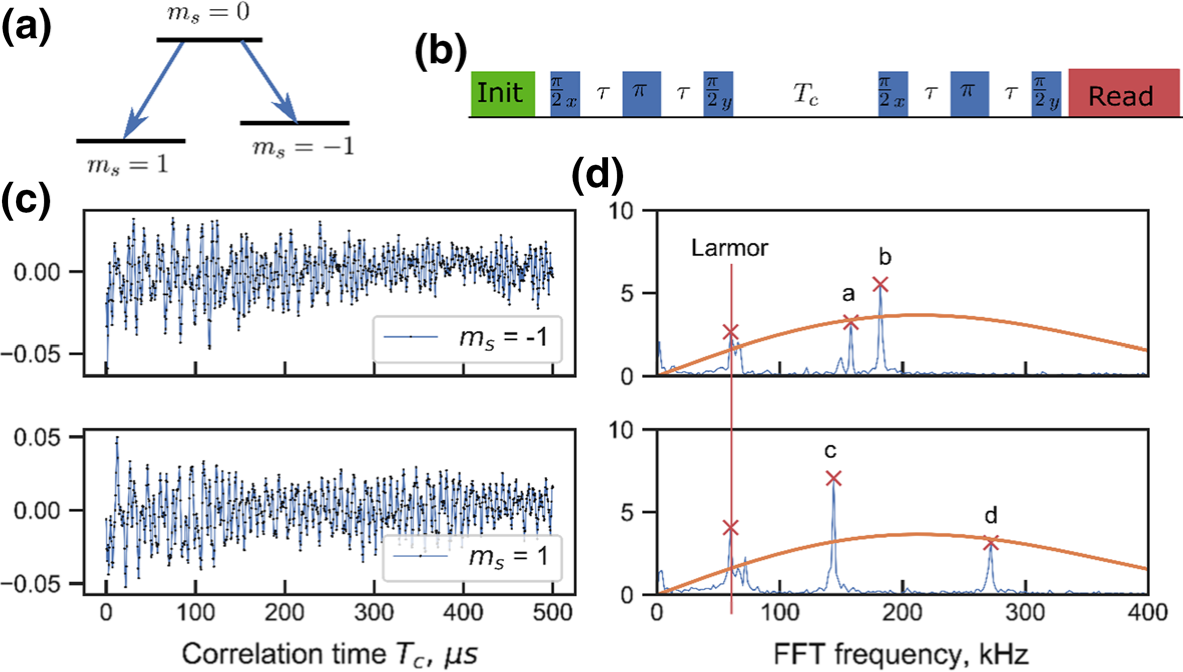
Correlation spectroscopy to resolve nuclear bath hyperfine spectra. **a** Ground-state triple energy level scheme. **b** Correlation protocol pulse sequence. It consists of two Hahn echo sequences interspaced with long correlation time ${T}_{\mathrm{c}}$. The $\pi /2$ pulses at the end of each HE are 90° phase shifted. **c** Contrast data output of the measurement protocol. The results from ${m}_{\mathrm{s}} = 1$ and ${m}_{\mathrm{s}} = -1$ subdomain show different beating features. **d** Absolute value of FFT taken to the data at **c**, showing the spectral features of the bath interacting with the central spin. The component at approximately 60 kHz is the bare nuclear Larmor frequency, while other peaks correspond to the eigen frequencies in ${m}_{\mathrm{s}}=1$ and ${m}_{\mathrm{s}}=-1$ subdomains for individual nuclear spins. The solid orange curve marks the filter function of a single HE sequence, for comparison with the resolution gained in the correlation sequence

Several schemes have been proposed and demonstrated to further narrow the bandwidth and to perform high resolution spectroscopy. All of them rely on correlation measurements. In the present paper, we use a standard 5-pulse ESEEM ESR sequence with a final *π*/2 pulse for optical readout of *z* spin orientation shown in Fig. 3b. The pulse sequence consists, as shown in Fig. 3, of a multipulse sequence, however, is subdivided into two equal sensing periods that are separated by an incremented free evolution period *T*_c_. Since the multipulse sequence is phase sensitive, constructive or destructive interference occurs between the two sequences, depending on whether the free evolution period between the two multipulse blocks is an integer multiple of the Larmor period of the nuclear spin to be detected. The final signal thus oscillates as [1]

$$P\left(t\right)\approx {\mathrm{sin}}^{2}{A}_{zx}^{i}\tau (\mathrm{cos}{\omega }_{\mathrm{L}}{T}_{\mathrm{c}}+\mathrm{cos}{\omega }_{\pm 1}^{i}{T}_{\mathrm{c}}).$$

Here, ${A}_{zx}\tau$ is the maximum phase that can be accumulated during either of the two multi-pulse sequences and ${\omega }_{\mathrm{L}}$ and ${\omega }_{\pm 1}$ are the nuclear transition frequencies in ${m}_{\mathrm{s}}=0$ and ${m}_{\mathrm{s}}=\pm 1$ subdomains. The approximate equation is valid for small signals with $\mathrm{sin}\alpha \approx \alpha$. Since the signal is encoded in the population of the electron spin during the ${T}_{\mathrm{c}}$, relaxation is no longer determined by *T*_2_ but rather *T*_1_ with a concomitant much longer *T*_c_. By this, a resolution of Δ*ν* ∼ 1/*T*_1_ is achievable. This resolution can be further increased using long-lived memory qubits, such that resolution enhancement of two to three orders of magnitude is possible [27].

In the following, we use this resolution enhancement to unravel the hyperfine coupling underlying the complex response of Fig. 2d. To this end, we use two Hahn echo sequences for phase acquisition separated by *T*_c_ (see Fig. 3b). We repeat the sequence for the two allowed EPR transition of the system, namely ${m}_{\mathrm{s}}=0$ to 1 and ${m}_{\mathrm{s}}=0$ to − 1 (see Fig. 3a). The correlation experiment traces, i.e., signals as a function of *T*_c_, are shown in Fig. 3c for both transitions. Figure 3d finally shows the Fourier transformations of both signals. Both traces show clearly resolved spectral components, stemming from the coupling of the electron spin to a small number of nuclear spins with frequencies in a range of around 50 to roughly 300 kHz. For comparison, Fig. 3d also shows the spectral resolution we would get if we would only use a single Hahn Echo sequence demonstrating the dramatic improvement in resolution gained by the correlation sequence. Specifically, the obtained FWHM was on the order of 2 kHz, at a *T*_c_ of 500 μs. We would like to add that the upper limit of *T*_c_ is limited by *T*_1_ of the electron spin which is around a microsecond at room temperature but extends to be 1 h at *T* < 10 K [28].

## Reconstruction of the Nuclear Environment

**Fig. 4:**
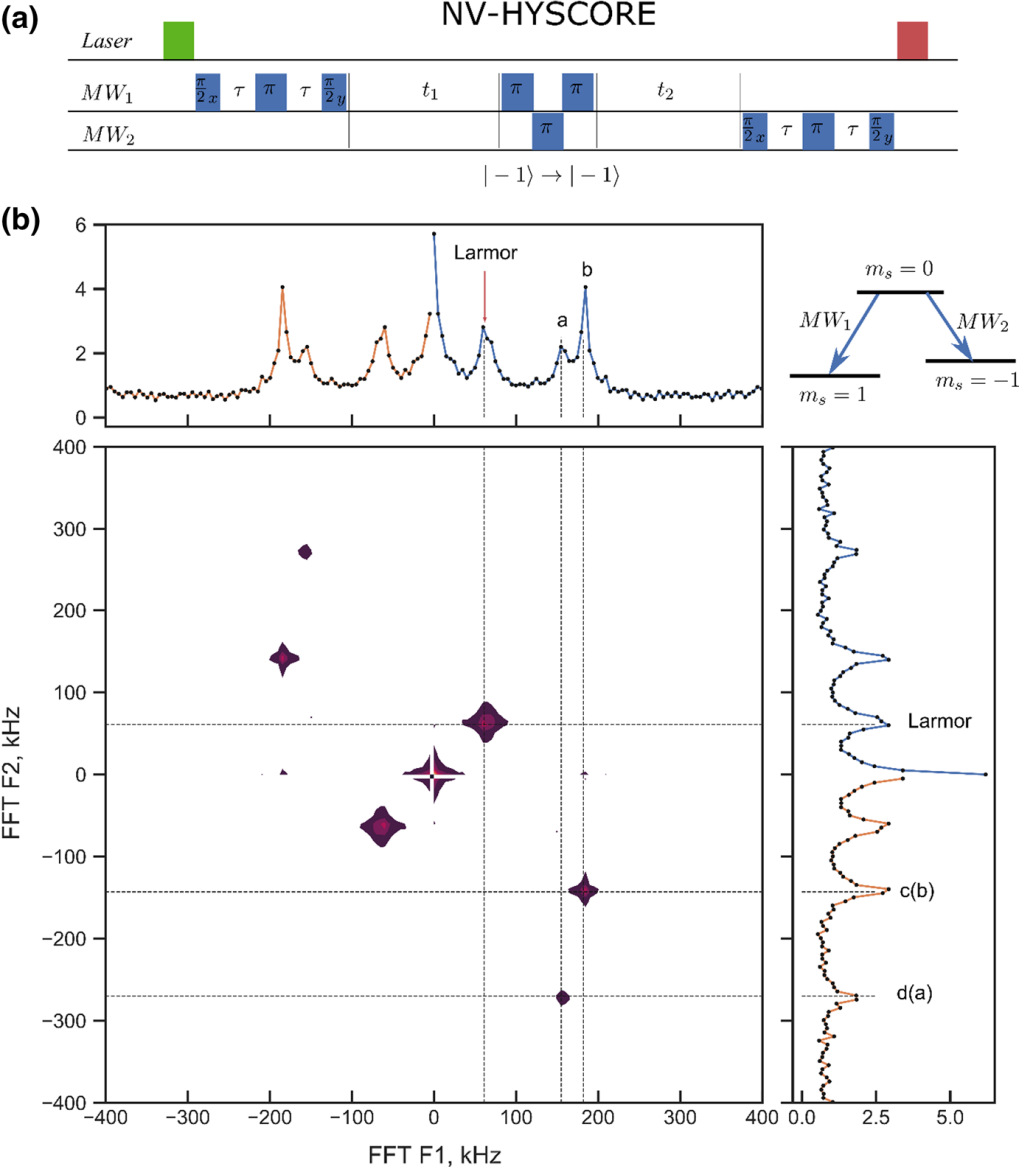
2D correlation spectroscopy (HYSCORE) sequence performed with NV center. **a** Pulse sequence used for the experiment. **b** 2D FFT of the obtained time domain data set. The peaks from *m*_s_ = − 1 are clearly correlated with peaks from the *m*_s_ = 1 electron sublevel. This resolves the uncertainty of attributing spectral lines to individual nuclei from the 1D correlation measurements shown in Fig. 3

In the correlation spectra in Fig. 3d, we measure four peaks which we attribute to the hyperfine coupling of nuclear spins. The two traces in the figure correspond to the *m*_s_ = 0 to *m*_s_ =  + 1 (upper trace) and *m*_s_ = 0 and *m*_s_ = − 1 ESR transition. We use the fact that the positions of these peaks in the correlation spectra can be described in secular approximation. As a consequence, we expect to detect signals at frequencies ${\omega }_{\mathrm{L} }\mathrm{and} {\omega }_{\pm 1}=\sqrt{{\left({\omega }_{\mathrm{L}}\pm {A}_{zz}\right)}^{2}+{A}_{zx}^{2}}$ [1]. The peak at around 60 kHz is identical in both traces, i.e., marks the position of the nuclear Larmor frequency ${\omega }_{\mathrm{L}}$. As each trace contains two additional peaks (i.e., peaks a, b, c, d), we conclude that we resolve the hyperfine coupling of two nuclei. In total, we have four unknown parameters (${A}_{zz}^{\mathrm{nuc1,2}}, {A}_{zx}^{\mathrm{nuc1,2}}$) but only two data sets. The remaining uncertainty is which peaks we should attribute to which nuclear spin in the two traces. To solve this uncertainty, we additionally perform a two-dimensional scan of the correlation time *T*_c_, by splitting it into *t*_1_ and *t*_2_ (see Fig. 4a). *t*_1,2_ are separated by a three *π*-pulses to convert population between *m*_S_ = − 1 and *m*_S_ = 1, thus realizing a HYSCORE [1] sequence for spin *S* = 1 systems. The idea behind this sequence is that it allows to correlate nuclear coherences for *t*_1_ in *m*_S_ = − 1 with *m*_S_ = 1 for a time interval *t*_2_. After Fourier transformation, this yields correlated NMR spectral features in *m*_S_ = − 1 and *m*_S_ = 1. This resolves dense 1D correlation spectra we obtain nuclear spin couplings [1]. After determining the coupling parameters for the two nuclear spins seen from the correlation spectra we fit the ESSEM features in Fig. 2c with an analytical model in the approximation of non-interacting nuclear spins. To find unknown hyperfine coupling in the ESSEM spectra, we iteratively add additional nuclear spins, and fit the numerical model to the ESEEM data of Fig. 2c. For this fit, new spins are added with an initial guess for their hyperfine coupling *A*_*zz*_ = *A*_*zx*_ = 1 kHz. The two strongly coupled spins, found from correlation spectra, were fixed throughout the iterative search. The best fit of the data in Fig. 2c is achieved when we add four nuclear spins. As a result, we derive the set of nuclear spins presented in Table 1.

**Table 1:** Hyperfine coupling data derived from the analysis of the correlation spectroscopy data in Fig. 3d

Spin #	1	2
*A*_*zz*_ (kHz)	− 204.0 (5)	51.5 (5)
*A*_*zx*_ (kHz)	64.4 (5)	143.6 (5)

## Methods

In the section below we briefly summarize the equations used to fit the HE data as well as hyperfine coupling data derived from those fits.

### Two Pulse ESEEM Simulation (Hahn Echo)

To efficiently fit the experimental data with the model we used a simplified analytical expression, developed in Rowan et al. [30], where for *N* spins, with the Hamiltonian in secular approximation expressed in the eigenstates of electron spin subspace Hamiltonian

$${H}_{i}=|0\rangle \langle 0|\overrightarrow{{\omega }_{0}^{i}}\cdot \overrightarrow{{I}_{i}}+\left|\pm 1\right.\rangle \left.\langle \pm 1\right|{\overrightarrow{{\omega }^{i}}}_{\pm 1}\cdot \overrightarrow{{I}_{i}}$$

we could obtain the ESEEM signal of two pulse (Hahn echo) sequence as:

$$V\left(\tau \right)={\prod }_{i=1}^{N}{V}_{i}(\tau )$$

$${V}_{i}=V\left(0\right)[1-2{\left(\frac{|\overrightarrow{{\omega }_{0}^{i}}\times {\overrightarrow{{\omega }^{i}}}_{\pm 1}|}{{|\omega }_{0}^{i}{||\omega }_{\pm 1}^{i}|}\right)}^{2}{\mathrm{sin}}^{2}\left(\frac{{|\omega }_{0}^{i}|\tau }{2}\right){\mathrm{sin}}^{2}\left(\frac{|{\omega }_{\pm 1 }^{i}|\tau }{2}\right)]$$

### Correlation Spectroscopy Signal

Though the interpretation of FFT of the correlation spectroscopy is more intuitive, we provide here a solution of the signal shape for the 3 and 5 pulse ESEEM signal [1] (Ch10) for the case of the NV center

$$\langle {S}_{z}\rangle \approx {\mathrm{sin}}^{2}{A}_{zx}\tau \left(\mathrm{cos}{\omega }_{0}{T}_{\mathrm{c}}+\mathrm{cos}{\omega }_{\pm 1}{T}_{\mathrm{c}}\right)$$

### Two-Dimensional Correlation Spectroscopy HYSCORE

Our HYSCORE method is adapted from conventional ESR two-dimensional spectroscopy. We sweep the corresponding *t*_1_ and *t*_2_ times with exponential scaling which forms a geometrical progression [31] and reduces the number of points by a factor of 4 in comparison to a uniform spacing, used for Fig. 3c.

## Conclusion

**Fig. 5:**
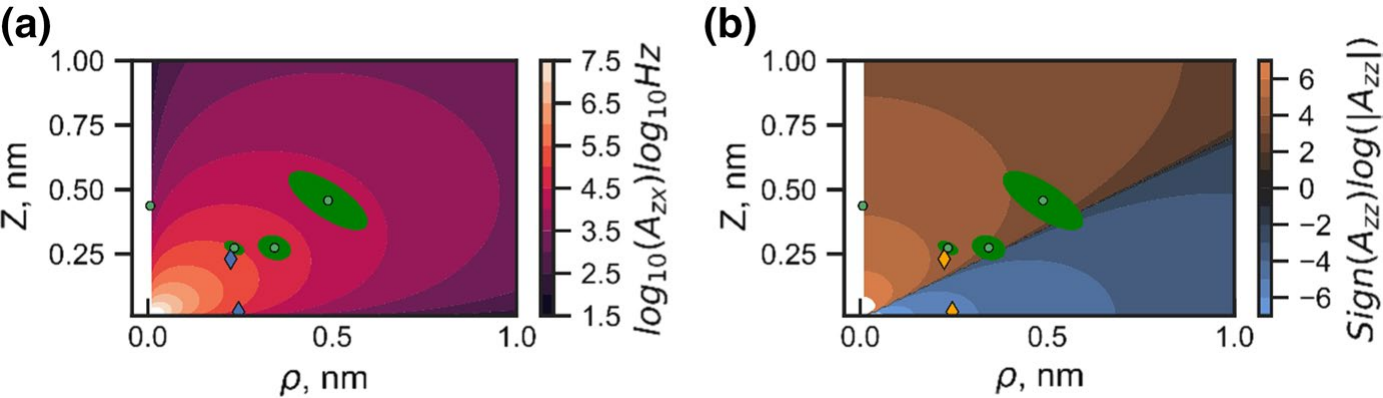
Spatial positions of the nuclei, obtained from the hyperfine spectra. Diamond markers specify strongly coupled nuclear spins inferred precisely from correlation spectroscopy (5 pulse ESSEM) and green dots are those which are added during the iterative fitting of the Hahn Echo 2 pulse ESEEM with ellipses determining the uncertainty of the fit. **a**, **b** *A*_*zx*_, *A*_*zz*_ as a function of radial in-plane distance *ρ* and axial position *z*

The identification and measurement of the hyperfine coupling of individual nuclear spins close to electron spin quantum bits is of major importance for their application in quantum technology. The combination of different methods like high resolution correlation spectroscopy and decoupling sequences as well as standard CW ESR spectra allows to identify these couplings across a wide range of coupling strengths. In this paper, we have made use of 1D and 2D correlation spectroscopy to spectrally resolve individual nuclear spins in an efficient way. Specifically for those nuclei which do show a hyperfine coupling of a few 10 kHz this method is useful, as it makes best use of the long spin relaxation times of the electron spin at temperatures of around and below 10 K. For the present case we have identified two ^13^C spins to couple to the NV electron spin with coupling strengths (*A*_*zz*_) of around − 200 and 60 kHz. In addition, we combined ESSEM and 1D and 2D correlation data to identify the hyperfine coupling tensor components of those nuclei. The NV center is a point defect, i.e., the spin density of the triplet electrons is almost entirely located at the three dangling bonds of the vacancy. As a result, the hyperfine coupling of the two nuclei is determined by their dipolar component. Hence, we can derive information on their location in the lattice. Using *A*_*zz*_ and *A*_*zx*_ we cannot determine the lattice position with atomic precision but give areas at which the spin is located [29]. The result is shown in Fig. 5.

The figure depicts the position of the two nuclei 1 and 2 whose hyperfine coupling is identified in the correlation experiment as a function of their radial as well as axial distance. It is apparent from the figure, that they are located significantly closer to the electron spin than the green dots, which mark those nuclei which have been added to fit the ESSEM spectra (see Table 2). Their hyperfine coupling can only be determined with a significantly larger error bar and hence their position is less precisely determined. Figure 4b shows *A*_*zz*_, i.e., the axial position of the nuclei. The nodal line represents the magic angle. While nuclei 1 and 2 do have close to identical in-plane distance *ρ*, their *A*_*zz*_ value are distinctly different. Nucleus 1 has a negative *A*_*zz*_ value and hence is located below the nodal line, nucleus 2 with a positive *A*_*zz*_ is above that line. Our method is thus very well suited to identify nuclei which on the one hand do show a coupling larger enough to allow for sub millisecond swap times of quantum states between an electron and a nuclear spin. Specifically, the *A*_*zz*_ value is responsible for the dephasing of the nuclei upon optical excitation of the electron spin. A small value, like for nucleus 2 yields maximum robustness of the nuclear spin quantum phase against optical excitation of the electron spin. It is this type of nuclear spins which will play a key role in future quantum networks.

**Table 2:** List of nuclei used to fit the ESEEM, XY-4 and correlation spectroscopy data

Spin #	3	4	5	6
*A*_*zz*_ (kHz)	6 (24)	49 (31)	4 (18)	76 (1)
*A*_*zx*_ (kHz)	55 (24)	101 (28)	16 (31)	1 (2)

Numbers in brackets are values for error
